# Idiotypic-susceptible Alzheimer’s disease: a clinically relevant, neurofibrillary tangle subtype

**DOI:** 10.1007/s00401-026-03013-6

**Published:** 2026-05-02

**Authors:** John L. Robinson, Helen Cai, Nicholas J. Loh, Omar Vazquez, Zahra Khodakaramimaghsoud, EunRan Suh, Vivianna M. Van Deerlin, Paul A. Yushkevich, Dawn Mechanic-Hamilton, Sharon X. Xie, Daniel T. Ohm, David A. Wolk, David J. Irwin, Jeffrey S. Phillips, Edward B. Lee

**Affiliations:** 1 Center for Neurodegenerative Disease Research (CNDR), Department of Pathology and Laboratory Medicine, Institute On Aging, University of Pennsylvania Perelman School of Medicine, Maloney Building, 3rd Floor, Philadelphia, PA 19104 USA; 2 Department of Biostatistics, Epidemiology and Informatics, University of Pennsylvania, Philadelphia, PA USA; 3 Department of Bioengineering, University of Pennsylvania, Philadelphia, PA USA; 4 Department of Radiology, University of Pennsylvania Perelman School of Medicine, Philadelphia, PA USA; 5 Department of Neurology, University of Pennsylvania, Philadelphia, PA USA

**Keywords:** Alzheimer’s disease, Non-amnestic, Tau, Neurofibrillary tangle, Subtype

## Abstract

Neurofibrillary tangles in Alzheimer’s disease (AD) stereotypically spread from the medial temporal lobe to association areas and then to idiotypic areas (i.e., primary motor, somatosensory, auditory, and visual). Previous studies have reported variable and clinically relevant tangle densities across the hippocampus and association cortices, but the idiotypic tangle burden is understudied. In this study, we measured tangle density using immunohistochemistry in three idiotypic cortices (primary motor, somatosensory, and visual), three association cortices (middle frontal, superior temporal, and inferior parietal), and two hippocampal sectors (CA1 and subiculum) in 144 cases with a high level of AD neuropathologic change. Clinical diagnoses included late-onset AD (LOAD, *n* = 50), early-onset AD (EOAD, *n* = 21), behavioral variant frontotemporal dementia (bvFTD, *n* = 19), corticobasal syndrome (CBS, *n* = 18), logopenic primary progressive aphasia (lvPPA, *n* = 21), and posterior cortical atrophy (PCA, *n* = 15). We algorithmically assigned cases outside the interquartile ranges of mean tangle ratios of association:hippocampal, idiotypic:association, and idiotypic:hippocampal to mutually exclusive subtypes: idiotypic-susceptible, associative-predominant, limbic-predominant, or typical Braak (for all remaining cases). Regional tangle burdens differentiated subtypes, while female sex, younger ages, and longer disease durations also influenced tangle severity. Compared to typical Braak cases, idiotypic-susceptible and associative-predominant cases exhibited shorter disease duration and younger age at death while limbic-predominant cases were older. The *MAPT* H1H1 haplotype also differed by subtype, being most prevalent in limbic-predominant and least common in idiotypic-susceptible and associative-predominant subtypes. Clinically, the idiotypic-susceptible subtype associated with CBS (56%), the associative-predominant subtype with bvFTD (53%), and the limbic-predominant subtype with LOAD (14%). The typical Braak subtype characterized 74–76% of amnestic AD cases and 32–53% of non-amnestic AD cases. Moreover, k-means clustering corroborated four clusters including the idiotypic-susceptible and associative-predominant patterns. Our results confirm previously described tau subtypes and describe an idiotypic-predominant subtype with clinical relevance and distinct demographic and genetic characteristics in AD.

**Supplementary Information:**

The online version contains supplementary material available at 10.1007/s00401-026-03013-6.

## Introduction

Braak and Braak’s staging of tau-positive neurofibrillary tangles (NFTs) in Alzheimer’s disease (AD) is based on both the distribution and severity of NFTs [4]. At end stage, tau is presumed to have spread from the entorhinal cortex to the hippocampus and into the association cortices such as the middle frontal cortex, superior temporal cortex, and inferior parietal cortex before affecting the idiotypic or primary cortices such as the primary motor cortex, primary somatosensory cortex, and primary visual cortex. This stereotypical progression is typically associated with increased NFT burden in areas affected earlier in the disease course as tau pathology progresses throughout the brain. As such, a typical Braak VI end-stage AD brain has an NFT burden that is greatest in the hippocampus, lower in the association cortices, and least in the idiotypic cortices. However, recent studies have highlighted considerable heterogeneity in Alzheimer’s disease, identifying distinct subtypes based on NFT or tau distributions. Murray et al. used post-mortem histology to define three subtypes: hippocampal-sparing, limbic-predominant and typical [19]. Vogel et al. used tau-PET in vivo imaging to define four patterns: limbic-predominant, medial temporal lobe-sparing, posterior, and lateral temporal [46]. Both studies found that tau subtypes are associated with different clinical characteristics, demographics, and disease progression. Recent work has repeated and extended these initial findings. In one study, compared to typical AD, hippocampal-sparing AD performed significantly worse in all cognitive domains at the time of initial impairment and declined significantly faster in memory, language, and globally [44]. Other studies concluded that AD subtypes have distinct atrophy patterns and network disruptions that correlate with clinical symptoms [7, 22, 27, 47].

Clinically, AD is a heterogeneous group of syndromes. Most patients have an amnestic profile, but language difficulties, decreases in visuospatial skills, movement impairments, and executive dysfunction also occur [26]. When these non-amnestic cognitive declines are prominent, patients are described as having atypical or non-amnestic AD. These non-amnestic syndromes include logopenic variant primary progressive aphasia (lvPPA), which is characterized by word-finding difficulties and is frequently neuropathologic AD [34], posterior cortical atrophy (PCA) characterized by visual-perceptual and -spatial difficulties that is also frequently neuropathologic AD, corticobasal syndrome (CBS) whose symptoms may include movement difficulty and poor coordination where neuropathologic AD may account for one-third of cases [35], and behavioral variant frontotemporal dementia (bvFTD), for which neuropathologic AD is a rare underlying pathology[23].

The typical pattern of NFTs strongly correlates with amnestic presentations of AD, but the association between other tau subtypes and non-amnestic presentations is less understood. Murray et al. reported that 75% of cases had a typical Braak subtype and this has been reproduced in other histological studies where it averages 72–85% in amnestic AD cohorts [15, 19, 23, 44, 47]. In non-amnestic AD studies, the typical Braak subtype is still prevalent (60–85%), but the hippocampal-sparing subtype is more frequent and the limbic-predominant subtype is less frequent compared to amnestic cohorts [15, 19, 23, 30, 44, 47]. The prevalence of the hippocampal-sparing subtype was initially reported at 9% in amnestic AD and 20% in non-amnestic AD [19]. In a follow-up study, it was reported at 10% in amnestic AD and 38% in non-amnestic AD [15]. In addition, a sub-analysis of cases with a progressive dysexecutive phenotype revealed a 47% prevalence of the hippocampal-sparing subtype. Other studies report the hippocampal-sparing prevalence at 4–7% in amnestic and 11–31% in non-amnestic AD [23, 30, 44]. In contrast, the limbic-predominant subtype is more common in amnestic AD compared to non-amnestic cases. In amnestic AD cohorts the limbic-predominant subtype prevalence varies from 11 to23% and is 0–5% in most non-amnestic cohorts[15, 19, 23, 30, 47] with one study reporting a prevalence of 24% [44].

While histological studies consistently report that most individuals have a typical Braak subtype, Vogel et al.’s tau PET imaging study reported greater variability. In that study, the typical Braak subtype, which they labeled as the limbic-predominant pattern, accounted for 33% of cases while the other three patterns—medial temporal lobe-sparing (i.e., hippocampal-sparing), posterior and lateral temporal—were almost as prevalent at 18%, 30%, and 19%, respectively [46]. Another tau PET imaging study assigned typical Braak, hippocampal-sparing and limbic-predominant subtypes but also included a fourth minimal atrophy subtype [7]. In this study of an amnestic AD cohort, the distribution was typical Braak (51%), hippocampal sparing (17%), limbic predominant (17%), and minimal atrophy (15%). Other studies have reported that the heterogeneity of tau appears in preclinical AD cases and may involve up to seven patterns [11, 48]. Together, these studies imply that there may be more tau subtypes than have been previously described.

In this study we histologically examined a relatively large cohort of cases that were diagnosed with a high level of AD neuropathologic change [18]. Cases included clinically diagnosed early-onset AD (EOAD) and late-onset AD (LOAD) as well as non-amnestic clinical variants including bvFTD, CBS, lvPPA, or PCA. We hypothesized that the distribution of tau subtypes would differ among the six clinical syndromes. We extend previous work by quantifying the density of NFTs in areas of the idiotypic cortex in addition to areas of the associative cortex and the hippocampal formation. Doing so allowed us to classify cases into typical Braak, idiotypic-susceptible, associative-predominant or limbic-predominant subtypes. We also report on other factors that have been shown to influence NFT burden such as female sex [5], disease duration [45], age [32], and *MAPT* H1H1 haplotype [19].

## Materials and methods

### Cohort selection

Cases studied in the Center for Neurodegenerative Disease Research brain bank are mainly obtained from referrals from the Hospital of the University of Pennsylvania neurology clinics or clinical observational studies. Demographic, clinical, and neuropathological data were obtained for all patients (accessed April 2025). All patients were diagnosed through consensus by expert clinicians from the Penn Frontotemporal Degeneration Center and Alzheimer’s Disease Research Center per published diagnostic criteria [2, 6, 10, 16, 17, 28]. Of 569 patients with a primary diagnosis of a high level of AD neuropathologic change according to NIA-AA criteria [18], 521 had a clinical diagnosis of AD or frontotemporal dementia; 500 had available *APOE* and *MAPT* genetic data. We next checked for rare secondary neuropathologies and excluded 14 cases with a secondary neuropathological diagnosis of progressive supranuclear palsy, frontotemporal lobar degeneration with TDP-43 positive inclusions, tauopathy unclassifiable, or other diagnosis. We then excluded 23 cases with a secondary clinical diagnosis of Parkinson’s disease or a psychiatric illness. Of the remaining 463 cases, we included all CBS (*n* = 18), all bvFTD (*n* = 19), all PCA (*n* = 15), and a convenience sample of lvPPA cases based on tissue availability (*n* = 21/32). For the clinical AD cases, all *TREM2* and *PSEN1* variants were excluded, and a convenience sample of sporadic EOAD (< 65 years old, *n* = 21/108) and sporadic LOAD (*n* = 50/238) cases were included (Table 1).

**Table 1:** Cohort characteristics

Group	LOAD (*n* = 50)	EOAD (*n* = 21)	bvFTD (*n* = 19)	CBS (*n* = 18)	lvPPA (*n* = 21)	PCA (*n* = 15)
n	50	21	19	18	21	15
Female^a^	64%	62%	47%	78%	43%	27%
Early onset	0%	100%	47%	94%	71%	79%
Disease duration	10 (5)	11 (3)	10 (5)	9 (3)	10 (4)	10 (4)
Age at death	84 (6)	66 (7)	74 (12)	66 (7)	69 (9)	68 (8)
*APOE* e4	72%	71%	58%	67%	71%	47%
*MAPT* H1H1	74%	52%	63%	56%	76%	73%
Other genetic	0%	0%	5%^b^	0%	10%^c^	13%^d^

LOAD, late-onset Alzheimer’s disease; EOAD, early-onset Alzheimer’s disease; bvFTD, behavioral variant frontotemporal dementia, CBS, corticobasal syndrome; lvPPA, logopenic primary progressive aphasia; PCA, posterior cortical atrophy

^a^Values are mean (SD) in years or %

^b^One case with a *TREM2* p.R62H HET genotype

^c^One case with a *TREM2* p.R47H HET genotype and one case with a *TREM2* p.R62H HET genotype

^d^One case with a *PSEN1* p.V259A genotype and one case with a *TREM2* p.R47H HET genotype

### Histological methods

Histological sections are routinely evaluated for neuropathology after immunohistochemical staining using PHF-1 (a gift from Dr. Peter Davies) at 1:3,000 dilution to detect phosphorylated tau deposits, NAB228 (generated in the CNDR) at 1:20,000 dilution with formic acid pretreatment to detect β-amyloid deposits, 1D3 (a gift from Dr. Manuela Neumann) at 1:300 dilution with formic acid pretreatment to detect phosphorylated TDP-43 deposits, and Syn303 (generated in the CNDR) at 1:30,000 dilution with formic acid pretreatment to detect a-synuclein inclusions. Visualization is with the VECTASTAIN ABC-HRP Kit and DAB Substrate Kit (VectorLabs, USA). Hematoxylin and eosin staining is also performed to detect neuron loss, gliosis, infarcts, and arteriolosclerosis.

18 regions are routinely stained, including middle frontal cortex, superior temporal cortex, angular gyrus, primary visual cortex, anterior cingulate, amygdala, entorhinal cortex, posterior hippocampus including CA1 and dentate gyrus, putamen, globus pallidus, thalamus, midbrain, substantia nigra, upper pons, locus coeruleus, medulla, and cerebellum including dentate nucleus. The level of Alzheimer’s disease neuropathologic change is assigned after staging for tangles with Braak stage, plaques with Amyloid phase, and neuritic plaques with CERAD per NIA-AA criteria [2]. All 144 cases were assigned a high level of Alzheimer’s disease, including 136 described as A3B3C3 and 8 described as A3B3C2.

Co-pathologies included cerebral amyloid angiopathy (CAA), limbic-predominant age-related TDP-43 encephalopathy neuropathologic change (LATE-NC), Lewy body disease (LBD), and cerebrovascular disease (CVD). CAA was evaluated for both severity and subtype [6]. LATE-NC was staged as 0–3 [4]. The distribution of Lewy bodies was described as amygdala-predominant, brainstem-predominant, transitional or diffuse [2]. CVD was reported as being low, intermediate, or high based on a modification of the VCING guidelines where intermediate is defined by the presence of any infarct or the presence of both moderate/severe arteriolosclerosis and CAA, and high is defined by the presence of any infarct with moderate/severe arteriolosclerosis and/or moderate/severe CAA [5].

### Neurofibrillary tangle subtyping

Tau immunohistochemistry was performed on three idiotypic cortical regions (primary motor cortex, primary somatosensory cortex, and primary visual cortex), three association cortical regions (middle frontal cortex, superior temporal cortex, and angular gyrus), and two hippocampal sectors (CA1 and subiculum).

Digital images were obtained using a P250 slide scanner (3DHistech) with a 10 × magnification, then analyzed using QuPath (version 0.5). Image analysis protocols were adapted from the Penn Digital Neuropathology Lab [1]. Each region of interest (ROI) was manually outlined with the polygon tool. For the association and idiotypic cortices, each ROI area averaged approximately 3 mm^2^, while for the hippocampal regions the average was 2 mm^2^. Within each ROI, a tiling and reduction script randomly placed 175 um^2^ tiles filling 30% of the ROI or a minimum of ten tiles. NFTs in each tile were manually counted with the point tool. NFT density was calculated by dividing NFT counts by total tile area and standardized to per mm^2^. 1,570 NFT densities were obtained including 397 ROIs with multiple independent counts to ensure accuracy (Pearson correlation coefficient 0.84). If multiple densities were available, a mean was calculated, leading to 1,138 NFT densities (2 primary visual cortex and 12 primary somatosensory cortex densities were unavailable).

NFT subtypes were defined by the ratios of the regional means (idiotypic, associative, or hippocampal), along with individual median area densities (Table 1) as described below. Subtyping algorithms were adapted from the Mayo Clinic [19]. Regional means were determined by averaging the individual area NFT densities for idiotypic cortex, association cortex, and hippocampus. The values used to determine cutoffs were derived from the interquartile ranges of the regional ratios and the individual median area densities of all clinical AD cases (*n* = 71). Four subtypes were assigned: idiotypic-susceptible, limbic-predominant, cortical-predominant or typical Braak. Idiotypic-susceptible was defined if the idiotypic:associative ratio was in the upper 25% and the idiotypic:hippocampus ratio was in the upper 25%, and two of the idiotypic densities were greater than median values (15 for primary motor cortex, 18 for primary somatosensory cortex, and 10 for primary visual cortex). Limbic-predominant was defined if the hippocampus:associative ratio was in the upper 25% and both hippocampal densities were greater than median values (133 for CA1 density, and 65 for subiculum density). Associative-predominant was defined if the associative:hippocampus ratio was in the upper 25% and two of the associative densities were greater than median values (38 for middle frontal cortex, 52 for angular gyrus, and 57 for superior temporal cortex). The remaining cases were defined as typical Braak.

###  % area quantification

β-amyloid was quantified on two idiotypic cortical regions (primary motor cortex and primary visual cortex), two association cortical regions (middle frontal cortex and angular gyrus), and two hippocampal sectors (CA1 and subiculum) for a subset of the cohort representing five cases from each tau subtype.

Digital images were obtained using a P250 slide scanner (3DHistech) with a 10 × magnification and then analyzed using QuPath (version 0.5). Each region of interest (ROI) was manually outlined with the polygon tool. A positive pixel thresholder was set for the DAB channel using a moderate resolution, a Gaussian prefilter and a smoothing sigma of 0.5. The % β-amyloid was obtained from the thresholded area/the total ROI area.

### Clustering analysis

Unsupervised clustering was performed using the K-means algorithm in Python using pandas, scikit-learn, and scipy.stats and in R using the dicer (Diverse Cluster Ensemble in R) and mice (Multivariate Imputation by Chained Equations) packages. Prior to clustering, all regional tau measures were standardized using z-score normalization (mean = 0, standard deviation = 1) to ensure equal weighting across regions with different scales. The optimal number of clusters was determined using two complementary approaches: the elbow method and silhouette analysis. For the elbow method, within-cluster sum of squares (inertia) was computed for K = 1 to 10 clusters. For silhouette analysis, silhouette scores were calculated for K = 2 to 9 clusters to assess cluster separation quality. Both methods confirmed an optimal value of 4. Because 12 primary somatosensory and 2 primary occipital areas had missing densities, we used multivariate imputation by chained equations with predictive mean matching using the mice package to generate 50 imputed datasets. K-means clustering was done with K = 4 on each of the 50 imputed datasets. We then used a cluster-based similarity partitioning algorithm (CSPA) to yield a single set which was used to assign each participant to one of the four clusters.

### Statistical analysis

All statistical analysis including linear regression was done using R 4.5.0. Pairwise comparisons between subtype NFT densities were done using the Mann–Whitney tests. Demographic variable comparisons were done using t-tests. Correlations between subtypes and clusters were computed using Pearson’s correlation coefficients. Regional β-amyloid measures and NFT densities were compared using t-tests. Correlations b, as were correlations statistical tests were two-sided. Statistical significance was set at the 0.05 level.

### Genotyping

Genotyping was available for all cases. Each sample was genotyped from frozen brain tissue using previously described methods [43]. In addition to *MAPT* and *APOE* genotypes, *APP*, *PSEN1,* and *TREM2* genotypes were obtained. Five variants in *PSEN1* or *TREM2* were noted: *PSEN1* V259A in one case, *TREM2* R47H heterozygous in two cases, and *TREM2* R62H heterozygous in two cases.

## Results

We examined the NFT burden in a cohort of 144 individuals with a high level of AD neuropathologic change and either amnestic or non-amnestic clinical presentations (Table 1). Amnestic cases included patients with EOAD and LOAD. Non-amnestic cases included patients with bvFTD, CBS, lvPPA, and PCA. The majority of non-amnestic cases were early onset, except for the bvFTD group. Males and females were well represented in all groups, but CBS cases were more likely to be female and PCA cases were more likely to be male (χ^2^, *p* = 0.01). Genetically, most cases were sporadic and both the *APOE* e4 allele and *MAPT* H1H1 haplotype were common.

**Fig. 1:**
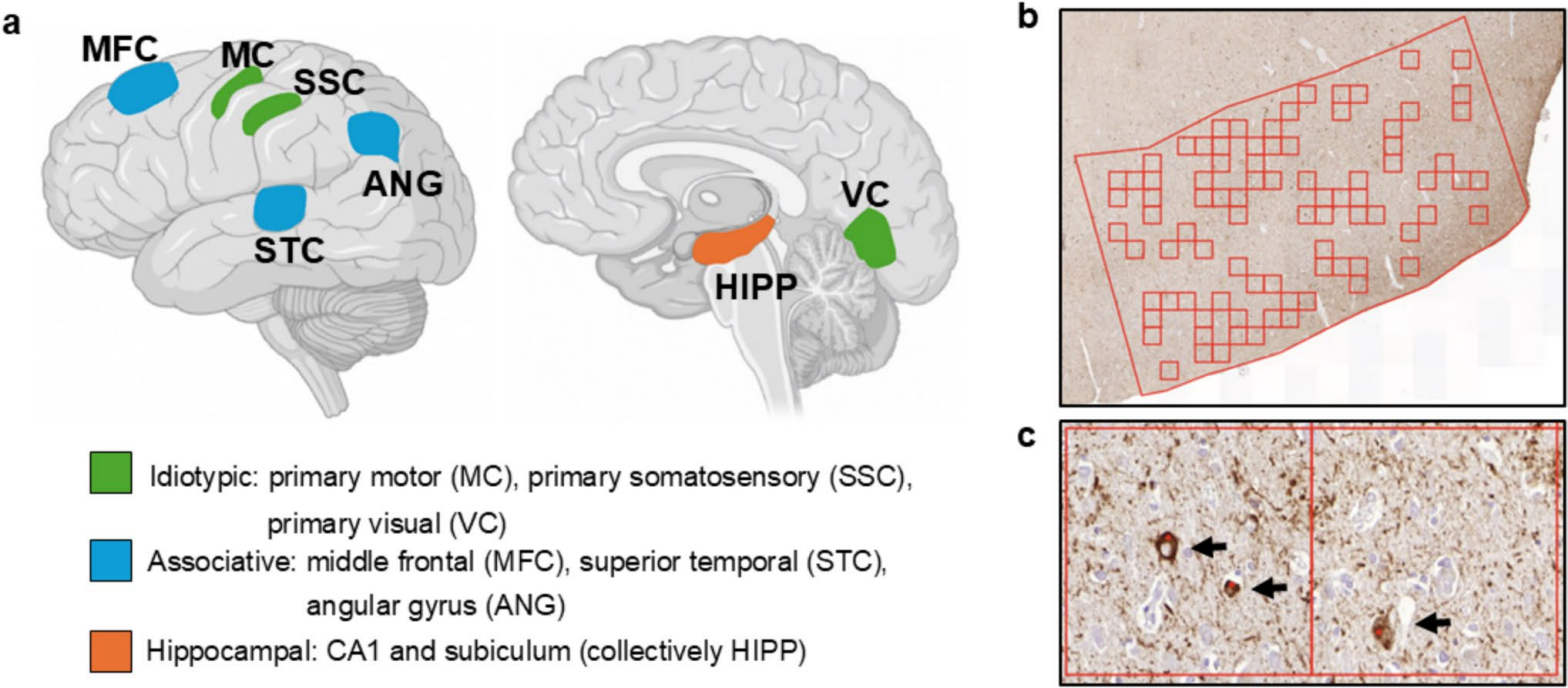
Regions sampled and NFT counting methods.** a** Highlighted regions correspond to areas sampled at autopsy. **b** Representative region of interest with 175 um^2^ tiles. **c** Manual quantification of NFTs (black arrows). NFT densities used in this study are counts/tile area, normalized to counts/mm^2^. *Illustrations adapted from Allen Brain Atlas generated with BioRender*

Digital microscopy was used to assess the NFT density in multiple regions, each of which was chosen to represent idiotypic cortex, associative cortex, and hippocampal subfields (Fig. 1). Regional idiotypic cortex, associative cortex, and hippocampal averages were obtained from individual area NFT densities. Most regional NFT densities were similar with some notable differences. In the hippocampus, the CA1 densities were usually higher than the subiculum densities. In idiotypic cortex, the primary motor and primary somatosensory densities were usually higher than the primary visual densities.

Normalized regional ratios of tau burden were calculated to assign each case into one of four patterns or subtypes (see methods for subtype criteria) corresponding to typical Braak, idiotypic-susceptible, associative-predominant and limbic-predominant (Table 2). The majority of cases had a typical Braak distribution of NFTs (n = 87, 60%). These are cases with a severe hippocampal burden, especially in CA1, a moderate associative cortex burden, and a mild idiotypic burden. 23 cases were classified as idiotypic-susceptible when the NFT densities were relatively low in the hippocampus subfields and associative cortex in contrast to a higher burden in the idiotypic cortices, especially the primary motor and primary somatosensory cortices. 24 cases were classified as associative-predominant when the hippocampus burden was low, but the associative cortex burden was high. Finally, 10 cases were classified as limbic-predominant when the tangles were high in the hippocampus subfields and low in the associative cortices. Regional measures were similar whether the left or right hemisphere was sampled (Supplemental Table S1).

**Table 2:** Subtype characteristics

	Typical Braak (*n* = 87)	Idiotypic-susceptible (*n* = 23)	Associative-predominant (*n* = 24)	Limbic-predominant (*n* = 10)	Idiotypic vs Braak^d^	Associative vs Braak^d^	Limbic vs Braak^d^
Regional ratios^a,b^							
Associative:hippocampal	0.33 (0.23)	0.49 (0.22)	0.77 (0.44)	0.11 (0.08)	** < 0.001**	** < 0.001**	** < 0.001**
Idiotypic:associative	0.58 (0.14)	0.96 (0.17)	0.34 (0.07)	0.69 (0.50)	** < 0.001**	** < 0.001**	0.188
Idiotypic:hippocampal	0.34 (0.10)	0.92 (0.14)	0.64 (0.17)	0.23 (0.06)	** < 0.001**	** < 0.001**	**0.013**
Tangle densities^c^							
CA1	140 (105–170)	90 (79–99)	87 (79–90)	171 (160–216)	** < 0.001**	** < 0.001**	**0.009**
Subiculum	65 (46–110)	43 (30–52)	46 (43–53)	88 (77–100)	** < 0.001**	**0.002**	0.217
Superior temporal	60 (45–76)	44 (36–51)	84 (70–102)	45 (39–50)	**0.001**	** < 0.001**	**0.034**
Middle frontal	49 (30–71)	45 (33–52)	74 (60–90)	15 (8–25)	0.260	** < 0.001**	** < 0.001**
Angular gyrus	55 (40–68)	55 (43–63)	69 (63–84)	22 (15–30)	0.933	** < 0.001**	**0.002**
Primary motor	16 (10–29)	32 (26–44)	19 (11–23)	11 (7–18)	** < 0.001**	0.833	0.252
Primary somatosensory	18 (11–26)	32 (25–45)	22 (12–30)	16 (7–22)	** < 0.001**	0.359	0.383
Primary visual	11 (5–18)	15 (8–18)	14 (6–22)	11 (4–17)	0.321	0.501	0.790
Demographics							
Age at onset	65 (10)	58 (10)	61 (10)	72 (7)	0.123	0.042	0.001
Disease duration	11 (7)	8 (7)	9 (4)	13 (8)	**0.008**	**0.037**	0.268
Age at death	76 (10)	69 (10)	68 (12)	85 (8)	**0.023**	**0.010**	**0.003**
Female	50 (57%)	13 (57%)	12 (50%)	6 (60%)	0.937	0.528	0.886
Non-amnestic	34 (39%)	18 (78%)	18 (75%)	3 (30%)	** < 0.001**	**0.001**	0.585
Co-pathology^e^							
CAA severity							
None	18 (21%)	7 (30%)	5 (21%)	1 (10%)			
Mild	15 (17%)	4 (17%)	4 (17%)	1 (10%)			
Moderate	20 (23%)	3 (13%)	5 (21%)	3 (30%)	0.410	0.970	0.235
Severe	34 (39%)	9 (39%)	10 (42%)	5 (50%)			
CAA subtype							
Type 1	24 (28%)	5 (22%)	7 (29%)	3 (30%)			
Type 2	45 (52%)	11 (48%)	12 (50%)	6 (60%)	0.746	0.884	0.640
LATE-NC							
None	44 (51%)	20 (87%)	18 (75%)	5 (50%)			
Stage 1	9 (10%)	0 (0%)	2 (8%)	0 (0%)			
Stage 2	31 (36%)	3 (13%)	1 (4%)	4 (40%)	**0.005**	**0.021**	0.545
Stage 3	3 (3%)	0 (0%)	3 (12%)	1 (10%)			
LBD distribution							
None	43 (49%)	11 (48%)	12 (50%)	3 (30%)			
Brainstem	6 (7%)	0 (0%)	0 (0%)	0 (0%)			
Amygdala	23 (26%)	6 (26%)	7 (29%)	3 (30%)	0.481	0.593	0.131
Limbic	8 (9%)	2 (9%)	3 (13%)	2 (20%)			
Diffuse	7 (8%)	4 (17%)	2 (8%)	2 (20%)			
CVD							
Low	60 (69%)	17 (69%)	14 (58%)	6 (60%)			
Intermediate	17 (20%)	5 (22%)	9 (38%)	3 (30%)	0.957	0.359	0.610
High	10 (11%)	2 (9%)	1 (4%)	1 (10%)			
Genotype							
APOE e4	63 (72%)	12 (52%)	13 (54%)	8 (80%)	0.093	0.121	0.603
* MAPT* H1H1	62 (71%)	10 (43%)	15 (62%)	10 (100%)	** < 0.001**	**0.001**	** < 0.001**
Other genetic	5^f^ (6%)	0 (0%)	0 (0%)	0 (0%)	**0.024**	**0.024**	**0.024**

*p*-values < 0.05 are highlighted in bold

CAA, cerebral amyloid angiopathy; LATE-NC, limbic-predominant age-related TDP-43 encephalopathy neuropathologic change; LBD, Lewy body disease; CVD, cerebrovascular disease; APOE, apolipoprotein E; MAPT, microtubule-associated protein tau

^a^Regional ratios are normalized from observed ratios in the clinical AD cases

^b^Variables are mean (SD) or %

^c^Densities are median (IQR)

^d^*p*-value is from t-test for mean values or Mann–Whitney for median values

^e^*p*-value is from t-test for mean values comparing moderate/severe to none/low for CAA and LATE-NC or amygdala/limbic/diffuse to none/brainstem for LBD

^f^Two cases with a *TREM2* p.R62H HET genotype, two with a *TREM2* p.R47H HET genotype, and one with a *PSEN1* p.V259A genotype

The subtypes differed by demographics, prevalence of co-morbid pathology and genetics (Table 2). Compared to those with a typical Braak subtype, patients with a limbic-predominant subtype were older at death, while those with an associative-predominant or idiotypic-susceptible subtype were younger. Disease duration was shorter for both the associative-predominant and idiotypic-susceptible subtypes. Non-amnestic clinical presentations were more likely in both the associative-predominant and idiotypic-susceptible subtypes. There was no difference by sex. We also examined the most common co-pathologies in Alzheimer’s disease including cerebral amyloid angiopathy (CAA), limbic-predominant age-related TDP-43 encephalopathy neuropathologic change (LATE-NC), Lewy body disease (LBD), and cerebrovascular disease (CVD). Neither CAA severity nor CAA type differed by subtype. LATE-NC was less frequent in the associative-predominant and idiotypic-susceptible subtypes. The prevalence of each LBD distribution, in general, did not differ between subtypes, although we note that a brainstem-predominant distribution was only observed in the typical Braak subtype. CVD prevalence was not different between subtypes. Genetically, the uncommon *TREM2* and *PSEN1* variants were only observed in the typical Braak subtype. The *APOE* e4 allele frequency did not differ between the subtypes. In contrast, the *MAPT* H1H1 haplotype was significantly more frequent in the limbic-predominant subtype, significantly less frequent in the associative-predominant subtype, and even less prevalent in the idiotypic-susceptible subtype.

We also explored if the plaque burden differed between subtypes. As there were no significant differences in Thal amyloid phase or CERAD neuritic plaque score, we determined β-amyloid burden as calculated by the percentage of area occupied in representative regional areas (middle frontal cortex and angular gyrus for associative cortex; motor cortex and primary visual cortex for idiotypic cortex; and CA1 and subiculum for limbic areas) in a subset of 20 cases. Overall, β-amyloid burden differed by region (Table 3) and was highest in the associative cortex (mean 8.6%), lower in the idiotypic cortex (mean 5.4%), and lowest in the limbic areas (mean 3.1%). However, there was no relationship between β-amyloid burden and NFT density in associative cortex, idiotypic cortex, or limbic regions. Moreover, regional β-amyloid burden was similar between subtypes (Table 4) except for a higher idiotypic cortex β-amyloid burden in limbic subtype cases compared to typical Braak subtype cases with a marginally significant p-value of 0.042 (without adjustment for multiple comparisons). From this analysis, we conclude that the regional tau burdens that define the tau subtypes are not significantly influenced by the β-amyloid burden in end-stage AD.

**Table 3:** Regional β-amyloid % and NFT density correlation

Region	β-Amyloid %	NFT density	Correlation
CA1^a^	4.0 (2.7)	141 (78)	0.27
Subiculum	2.3 (0.9)	69 (30)	0.01
Middle frontal	8.8 (2.4)	47 (27)	−0.21
Angular gyrus	8.5 (2.9)	57 (24)	0.19
Primary motor	5.3 (2.2)	20 (11)	0.01
Primary visual	5.6 (2.4)	14 (11)	−0.05

^a^Variables are mean (SD)

**Table 4:** β-Amyloid % area measurements by subtype

Region	Typical Braak (*n* = 5)	Idiotypic-susceptible (*n* = 5)	Associative-predominant (*n* = 5)	Limbic-predominant (*n* = 5)	Idiotypic vs Braak^b^	Associative vs Braak	Limbic vs Braak
CA1^a^	4.0 (4.1)	5.7 (2.9)	2.9 (0.9)	3.5 (1.2)	0.537	0.260	0.916
Subiculum	2.5 (0.7)	2.2 (1.1)	1.8 (1.1)	2.8 (0.8)			
Middle frontal	7.7 (1.9)	10.2 (1.7)	8.1 (2.4)	9.2 (3.1)	0.361	0.927	0.657
Angular gyrus	8.5 (3.1)	8.4 (2.5)	8.6 (2.8)	8.4 (4.2)			
Primary motor	5.2 (2.4)	4.2 (1.1)	4.8 (1.8)	7.0 (2.7)	0.901	0.401	**0.042**
Primary visual	4.1 (1.0)	5.4 (1.7)	6.8 (4.1)	6.1 (1.3)			

*p*-value < 0.05 is highlighted in bold

^a^Variables are mean (SD)

^b^t-test p-value using all regional β-amyloid % measures (*n* = 10 per group)

**Fig. 2:**
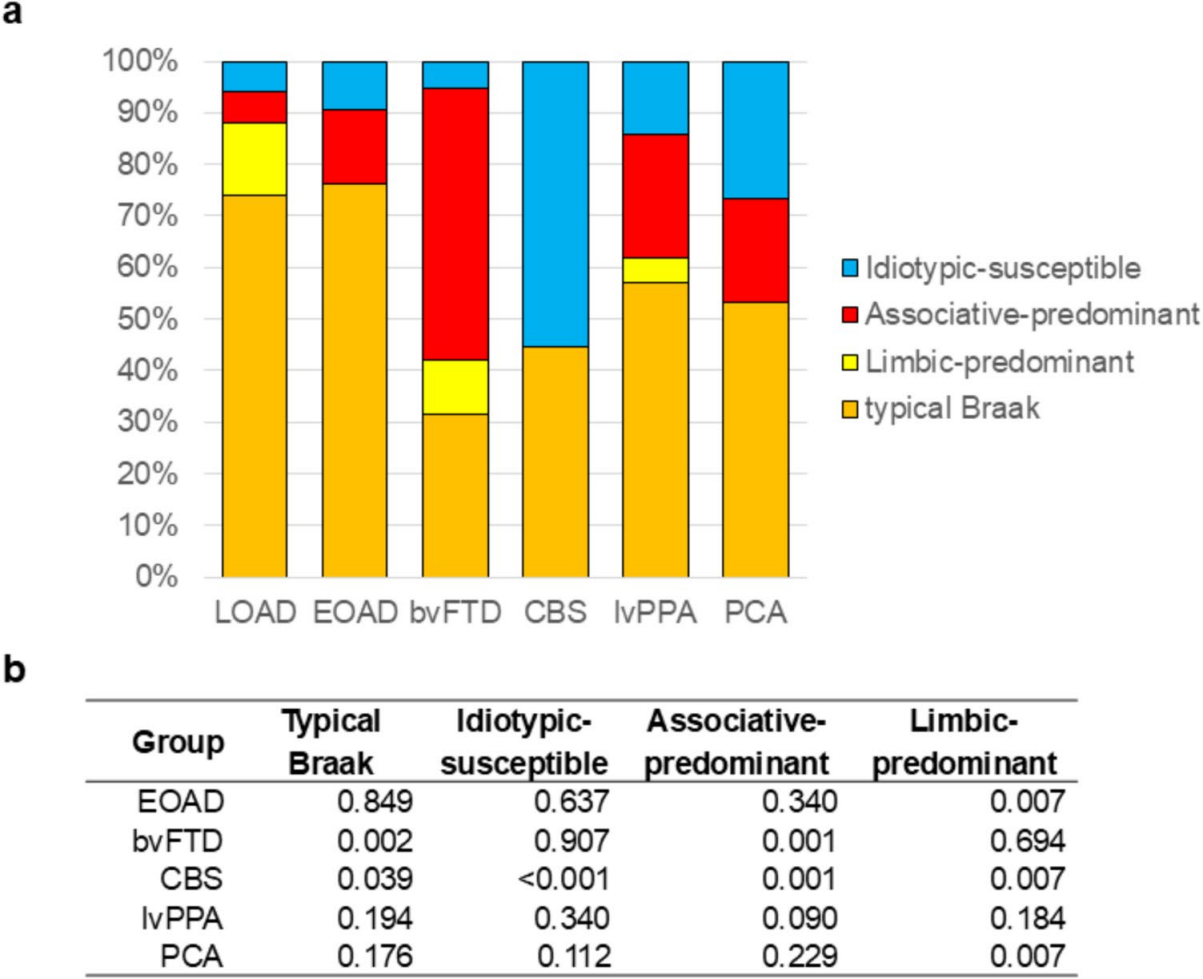
Subtype prevalence by clinical presentation.** a** Prevalence of the subtypes in each clinical group. **b** p-values comparing subtype prevalence in each clinical group relative to the LOAD group (t-test). Abbreviations: late-onset Alzheimer’s disease (LOAD), early-onset Alzheimer’s disease (EOAD), behavioral variant frontotemporal dementia (bvFTD), corticobasal syndrome (CBS), logopenic primary progressive aphasia (lvPPA), and posterior cortical atrophy (PCA)

Subtype prevalence differed significantly between clinical presentations (Fig. 2). The typical Braak subtype was observed in the majority (74%) of LOAD cases, in contrast with the limbic-predominant (14%), associative-predominant (6%), and idiotypic-susceptible (6%) subtypes. Similar to LOAD, EOAD cases were primarily the typical Braak subtype (76%), with few associative-predominant (14%) and idiotypic-susceptible (10%) subtypes and no limbic-predominant subtype observed. In the bvFTD group, the associative-predominant subtype represented the majority of cases (53%), while the typical Braak subtype accounted for only 32% of cases. In the CBS group, the most prevalent subtype was the idiotypic-susceptible pattern (56%), while the typical Braak subtype accounted for the remaining cases as both the limbic-predominant and associative-predominant subtypes were not observed. In the lvPPA group, just over half of the cases were typical Braak (57%) and the associative-predominant subtype was also common (24%). In the PCA group, about half of the cases were typical Braak (53%), and both the idiotypic-susceptible (27%) and associative-predominant (20%) subtypes were common, while the limbic-predominant subtype was not observed. Interestingly, the regional tau burden was similar within subtypes, between amnestic and non-amnestic AD cases (Supplemental Table S2).

**Fig. 3:**
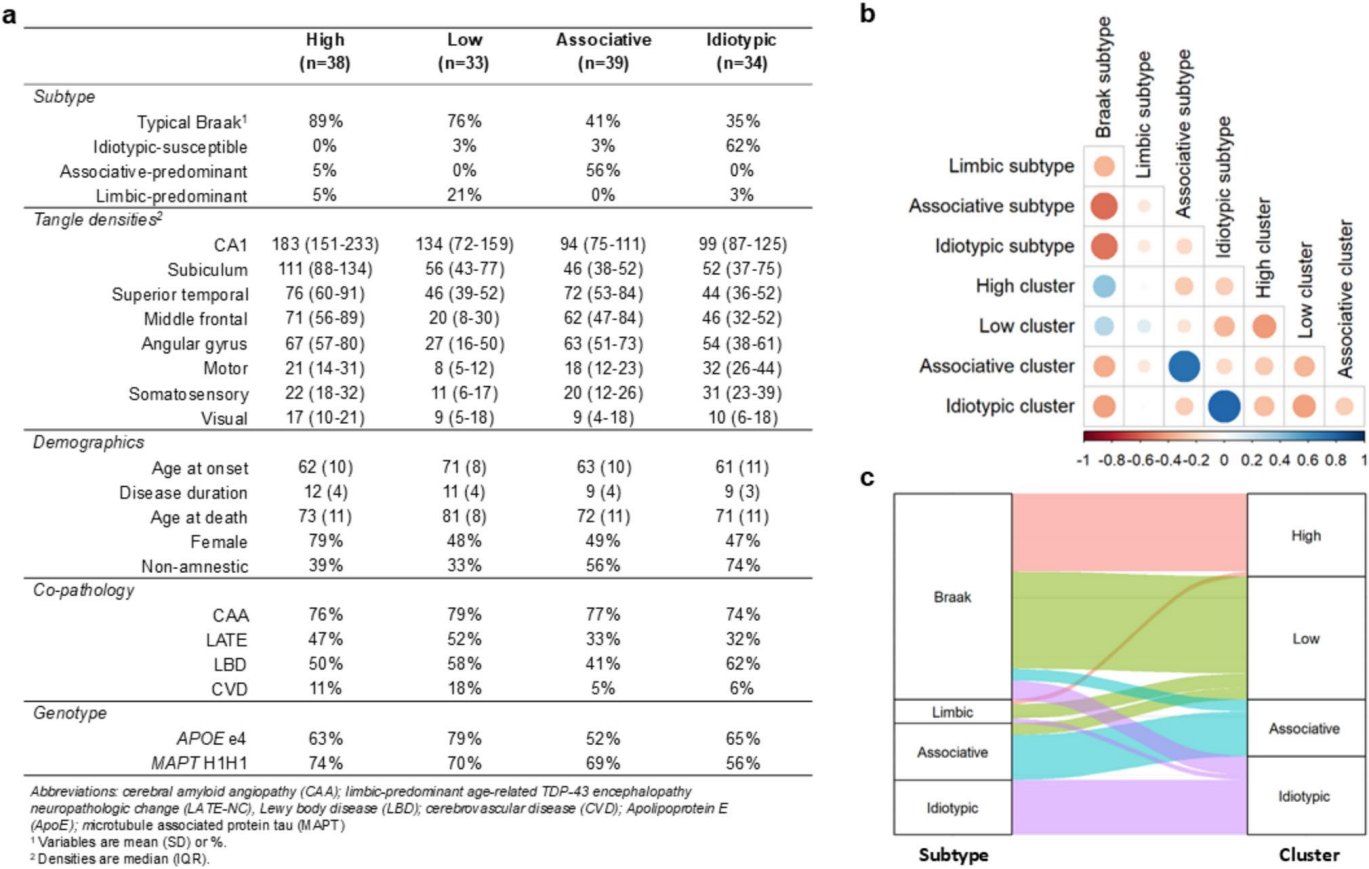
K-means clustering characteristics.** a** Cluster characteristics. **b** Correlation between subtypes and clusters (Pearson’s correlation coefficient). **c** Alluvial plot highlighting the composition of the subtype and cluster categories

Because the subtypes were defined by interquartile ranges of continuous NFT regional ratios [15] and the clinical relevance of tau subtypes to non-amnestic presentations of AD was questioned in another study [23], we also performed k-means clustering analysis as another independent approach to identify categorical subtypes (Fig. 3). Data-driven clustering resulted in four clusters which we designated as high, low, associative, and idiotypic based on the mean NFT distribution pattern of each. Overall, there was a strong correspondence between the clusters and the cutoff-derived subtypes (Fig. 3b). The high cluster was primarily composed of typical Braak subtype cases. Moreover, there was a strong concordance between the idiotypic k-means cluster and the previously defined idiotypic-susceptible subtype cases where only 2 of the 23 idiotypic-susceptible subtype cases clustered outside of the idiotypic cluster. There was also a strong concordance between the associative k-means cluster and the previously defined associative-predominant subtype cases where only 2 of the 24 associative-predominant subtype cases clustered outside of the associative cluster. Similarly, seven of the ten limbic-predominant subtype cases clustered in the low cluster. We conclude that while there are a few borderline cases when applying cutoffs along the “idio-associative-limbic” spectrum, the strong concordance between algorithmically defined-subtypes and k-means clusters suggests that these four groups may represent distinct distributional patterns of tauopathy in AD.

Finally, we built multiple linear regression models to assess non-subtype predictors of regional NFT variability (Table 5). Each model used the average regional hippocampal, associative or idiotypic NFT burden as the dependent variable. For these three models, we included demographic variables that have been reported to influence the NFT burden in previous studies, including sex [5], age [32], disease duration [29], non-amnestic syndromes [23], *MAPT* H1H1 genotype [19], and *APOE* e4 genotype [38]. Female sex and disease duration were associated with a higher hippocampal burden in all cases. This association was also seen in the subset of typical Braak subtype cases but not in the other subtypes. Younger age at death and a longer disease duration were associated with higher associative neocortex NFT burden. This association was also seen in the subset of cases with the typical Braak and idiotypic-susceptible subtypes but not in the other subtypes. A younger age at death and a longer disease duration were associated with higher idiotypic neocortex NFT density. A younger age at death was also a predictor of increased idiotypic neocortex burden in the typical Braak subtype but was not significant in the other subtypes. Interestingly, significant associations were not observed between specific regional NFT burden and a clinical presentation of non-amnestic dementia, *MAPT* H1H1 haplotype, or *APOE* e4 genotype.

**Table 5:** Predictors of tangle densities by tau subtype

	All cases		Typical Braak		Idiotypic-susceptible		Associative-predominant		Limbic-predominant	
	CE	*p*-value	CE^a^	*p*-value	CE	*p*-value	CE	*p*-value	CE	*p*-value
*Hippocampal NFTs*										
Female	19.3	**0.016**	25.4	**0.026**		0.477		0.235		0.138
Age at death^b^	−0.3	0.392		0.065		0.144		0.991		0.160
Disease duration^b^	3.9	** < 0.001**	4.4	**0.003**		0.849		0.340		0.216
Non-amnestic	−12.5	0.153		0.468		0.139		0.129		0.084
* MAPT* H1H1	15.8	0.058		0.509		0.356		0.598		–^c^
* APOE* e4	0.8	0.928		0.460		0.827		0.409		0.282
*Associative NFTs*										
Female	3.6	0.272	7.7	0.051		0.550		0.758		0.587
Age at death	−0.8	** < 0.001**	−0.8	** < 0.001**	−0.4	**0.036**		0.332		0.693
Disease duration	0.7	0.079		0.139		0.416		0.309		0.297
Non-amnestic	−0.1	0.983		0.527		0.245		0.998		0.912
* MAPT* H1H1	−1.3	0.706		0.769		0.682		0.837		–
* APOE* e4	−3.9	0.255		0.239		0.178		0.846		0.898
*Idiotypic NFTs*										
Female	1.5	0.303		0.201		0.948		0.856		0.443
Age at death	−0.3	** < 0.001**	−0.3	** < 0.001**		0.136		0.079		0.657
Disease duration	0.3	0.146		0.133		0.664		0.174		0.501
Non-amnestic	0.6	0.724		0.395		0.054		0.896		0.419
* MAPT* H1H1	−2.7	0.075		0.546		0.431		0.336		–
* APOE* e4	0.4	0.791		0.979		0.342		0.887		0.958

*p*-values < 0.05 are highlighted in bold

CE, coefficient estimates; APOE, apolipoprotein E; MAPT, microtubule-associated protein tau

^a^Coefficient estimates are shown if the p-value < 0.05

^b^Per year

^c^All limbic-predominant cases were *MAPT* H1H1

## Discussion

Our study adds to the diversity of NFT or tau patterns observed in brains with AD neuropathology. Recognition of AD subtypes helps clarify the observed heterogeneity in neuropathological AD which is otherwise glossed over when using consensus criteria to assign Braak stage VI to all end-stage AD cases. While the typical Braak subtype is the most common subtype in amnestic AD—accounting for 75% of cases in both early-onset and late-onset AD—it may only account for 50% of non-amnestic AD cases. By fully examining areas of the brain where NFTs accumulate late in the disease course, we describe the novel idiotypic-susceptible subtype. In comparison to the typical Braak pattern of NFTs, the idiotypic-susceptible subtype is primarily defined by an increased burden of primary motor and primary somatosensory NFTs, a reduced burden of hippocampal NFTs and a mildly reduced burden of associative cortex NFTs (Table 2). This pattern of NFTs was most common in CBS and PCA cases. We also report that the associative-predominant subtype, what others have described as hippocampal-sparing, is more common in bvFTD and lvPPA cases.

Previous studies have reported similar trends, although most did so without subtyping. A study specifically examining CBS cases reported more motor cortex tau and less temporal tau relative to amnestic AD cases [31]. Another study reported greater motor cortex NFTs in CBS and PCA cases [23]. Non-amnestic AD as an overall group exhibits more cortical tau than amnestic AD [15], while worse executive dysfunction and faster cognitive decline in early-onset cases are associated with a greater associative cortex to hippocampal NFT burden [37]. Similarly, in cases with aphasic AD, the ratio of associative cortex-to-entorhinal tangles was significantly higher [8, 20], while lvPPA specifically is associated with a trend for a higher ratio of temporoparietal-to-hippocampal NFT density [13]. The limbic-predominant subtype is more prevalent in late-onset AD [9, 19, 32, 44]. Imaging studies have reported that regional tau tracer uptake correlates with performance on domain-specific neuropsychological function, such as the medial temporal lobe and memory, the occipital lobe and visual spatial ability, and temporoparietal cortex and language performance [21]. At least two other imaging studies have examined non-amnestic AD cases and reported different neocortical distributions of tau that correlated with lvPPA, CBS, PCA, and bvFTD clinical diagnoses [24, 42]. Finally, two of the tau patterns Vogel et al. report are consistent with the clinicopathological correlations we describe for the idiotypic-susceptible and associative-predominant subtypes [46]. The posterior pattern was associated with visuospatial impairment and the medial temporal lobe-sparing was associated with dysexecutive symptoms. In summary, our results are consistent with and extend these prior studies.

There is relatively little known about the biological basis for clinical and neuropathologic heterogeneity in AD. While Braak stages are described as stepwise increments in the spread and severity of NFTs, in fact, certain stages may be more rapid or severe than others over the decades-long disease progression [3, 12] with age and genetics as contributing factors influencing spread. Previous studies have shown 10- to 100-fold differences in tau fibril seeding activity across brain regions in end-stage AD cases [40], raising the intriguing hypothesis that the biochemical properties of different tau seeds may influence the spread. In fact, tau activity changes significantly in early-stage AD [1], which is consistent with preclinical imaging studies that highlight tau heterogeneity early in the disease course [11, 39, 48]. We could not find a relationship between β-amyloid burden and the amount or distribution of tauopathy in AD. This is consistent with previous work where plaque burden did not differ between subtypes [3] and it implies that the tissue level processes that respond to β-amyloid plaques and give rise to NFTs are somewhat independent. However, sex, age, and disease duration all appear to significantly impact NFT density. Females tend to have more NFTs than males, especially in the hippocampus. Younger individuals have more associative-cortex NFTs than older individuals. Conversely, a longer disease duration results in a greater hippocampal burden. We conclude from this that while subtypes describe much of the observed heterogeneity in the AD brain, there are multiple factors that contribute to the regional NFT burden.

Genetically, *TREM2* risk variants and the *MAPT* H1H1 genotype have been associated with atypical clinical and neuropathologic subtypes of AD [14, 19]. In our study, the *MAPT* H1H1 genotype was less prevalent in idiotypic-susceptible and associative-predominant cases, and it was most prevalent in limbic-predominant cases. The latter two correlations were reported previously [19] but remain confounding. At least one study reports that the *MAPT* H1H1 genotype associates with a higher level of MAPT transcript expression in cortical tissue, but that the expression does not correlate with increased soluble or insoluble tau levels [41]. Others have reported that the typical Braak subtype is more likely in *APOE* e4 carriers, and that in LOAD cases, the limbic-predominant subtype is also more likely in *APOE* e4 carriers than non-carriers [19]. In contrast, our data are consistent with imaging studies that suggest that tau pathology may spread earlier in the disease course in *APOE* e4 carriers, and there is evidence that non-carriers “catch up” as the tau burden plateaus over time [25, 36].

There are several limitations to our study. First, digital quantitative pathology techniques differ between laboratories. Second, there is variability in the choice of antibodies or thioflavin S staining for detection of NFTs. However, both of these technical challenges likely affect the magnitude of quantification where relative differences are likely to be similar. Another limitation with our study is that for the tau density we capture the NFT density as this is readily quantifiable, but do not address the tau neurite burden even though this can be greater than tenfold higher than NFT burden [33]. In addition, there are other heterogeneities in the AD brain such as differences in density and morphology of amyloid plaques, and differences in microglial reactivity. Finally, while we were able to show clinical relevance by having a relatively large number of well-defined non-amnestic cases, overall, this is a small cohort due to the relative rarity of atypical AD clinical presentations. Thus, future studies should include larger cohorts to replicate our findings.

Our data support the hypothesis that end-stage AD is a syndrome of heterogeneous pathological subtypes, each with clinical relevance and associated demographic and genetic characteristics. Braak staging describes both the distribution and severity of NFTs in AD, but that single number minimizes the observed NFT variability in this disease. Our study continues and adds to previous work better characterizing NFT severity—and the factors that influence NFT burden including genetics, age, and female sex—which is increasingly important in the age of AD disease-modifying therapies.

## Supplementary Information

Below is the link to the electronic supplementary material.

Supplementary file1 (DOCX 20 KB)

## Data Availability

The data are available upon request.
