# Supplementary material for: Idiotypic-susceptible Alzheimer’s disease: a clinically relevant, neurofibrillary tangle subtype
Source: Acta Neuropathol. 2026 May 2;151(1):51. doi: 10.1007/s00401-026-03013-6 (PMC13135531; doi:10.1007/s00401-026-03013-6)
Supplement: Supplementary file 1 — Supplementary file1 (DOCX 20 KB) [file 401_2026_3013_MOESM1_ESM.docx]

**Supplemental data**

**Sup Table 1. Regional NFT burdens by hemisphere**

| **Subtype** | **Hemisphere** | **n** | **CA1**^1^ | **SUB** | **STC** | **MFC** | **ANG** | **MC** | **SEN** | **OCC** |
| --- | --- | --- | --- | --- | --- | --- | --- | --- | --- | --- |
| Typical Braak | Left | 47 | 135 (61) | 82 (53) | 58 (22) | 49 (25) | 52 (22) | 19 (10) | 20 (10) | 11 (8) |
|  | Right | 40 | 158 (68) | 84 (59) | 61 (26) | 54 (31) | 56 (22) | 21 (16) | 20 (13) | 13 (9) |
| Idiotypic-susceptible | Left | 12 | 93 (18) | 49 (21) | 44 (10) | 43 (18) | 57 (15) | 38 (14) | 39 (18) | 14 (11) |
|  | Right | 11 | 89 (23) | 37 (13) | 45 (13) | 43 (10) | 51 (15) | 30 (13) | 33 (15) | 14 (6) |
| Associative-predominant | Left | 12 | 89 (26) | 46 (15) | 82 (24) | 60 (20) | 77 (21) | 16 (7) | 20 (9) | 11 (8) |
|  | Right | 12 | 90 (22) | 53 (24) | 82 (31) | 91 (30) | 78 (36) | 20 (7) | 25 (16) | 16 (9) |
| Limbic-predominant | Left | 7 | 205 (101) | 85 (18) | 46 (16) | 20 (15) | 29 (24) | 18 (14) | 20 (10) | 11 (7) |
|  | Right | 3 | 195 (32) | 92 (13) | 40 (6) | 15 (15) | 27 (21) | 8 (4) | 8 (5) | 16 (23) |

^1^ Variables are mean (SD)

**Sup Table 2. Regional NFT burdens by subtype in amnestic and non-amnestic AD**

| **Subtype** | **Clinical** | **n** | **CA1**^1^ | **SUB** | **STC** | **MFC** | **ANG** | **MC** | **SEN** | **OCC** |
| --- | --- | --- | --- | --- | --- | --- | --- | --- | --- | --- |
| Typical Braak | Amnestic | 53 | 146 (71) | 84 (61) | 58 (22) | 48 (28) | 54 (23) | 18 (14) | 18 (10) | 11 (9) |
|  | Non-amnestic | 34 | 144 (55) | 81 (46) | 61 (26) | 56 (28) | 54 (21) | 23 (11) | 23 (14) | 13 (7) |
| Idiotypic-susceptible | Amnestic | 5 | 97 (16) | 65 (21) | 41 (8) | 39 (22) | 47 (19) | 44 (15) | 40 (29) | 18 (15) |
|  | Non-amnestic | 18 | 89 (21) | 38 (12) | 45 (12) | 44 (12) | 56 (14) | 32 (13) | 35 (12) | 13 (6) |
| Associative-predominant | Amnestic | 6 | 85 (23) | 40 (13) | 80 (24) | 69 (21) | 77 (24) | 18 (5) | 21 (9) | 13 (9) |
|  | Non-amnestic | 18 | 92 (24) | 53 (21) | 83 (29) | 78 (32) | 78 (31) | 18 (8) | 23 (14) | 14 (9) |
| Limbic-predominant | Amnestic | 7 | 169 (28) | 86 (18) | 45 (16) | 17 (10) | 28 (25) | 17 (14) | 18 (11) | 15 (14) |
|  | Non-amnestic | 3 | 279 (128) | 89 (11) | 41 (8) | 20 (24) | 30 (19) | 11 (10) | 12 (7) | 7 (7) |

^1^ Variables are mean (SD)
